# Hyaluronic Acid and Sodium Hypochlorite as Adjunctive Therapeutic Options for Patients with Periodontal Disease: A Systematic Review

**DOI:** 10.3390/biomedicines14020320

**Published:** 2026-01-30

**Authors:** Tomás Infante da Câmara, Francisca Abreu, Miguel Nunes Vasques, Ricardo Faria-Almeida, Honorato Ribeiro-Vidal

**Affiliations:** 1 Faculty of Dental Medicine, University of Porto, 4200-393 Porto, Portugal; 2 Specialization in Periodontology and Oral Implantology, Faculty of Dental Medicine, University of Porto, 4200-393 Porto, Portugal; 3 Associated Laboratory for Green Chemistry (LAQV) of the Network of Chemistry and Technology (REQUIMTE), 4050-342 Porto, Portugal

**Keywords:** cross-linked hyaluronic acid, sodium hypochlorite/amino acids, periodontal disease, non-surgical periodontal therapy, subgingival instrumentation, adjunctive treatment

## Abstract

**Background:** Periodontal disease is a chronic multifactorial inflammatory condition caused by dysbiosis of the dental biofilm, leading to destruction of the connective tissue attachment, alveolar bone resorption, and potentially tooth loss. Non-surgical periodontal therapy (NSPT), involving subgingival instrumentation, aims to restore periodontal health by reducing the probing pocket depth (PPD) and bleeding on probing (BOP) and by improving the clinical attachment level (CAL). The adjunctive use of chemical agents, such as sodium hypochlorite/amino acids (NaOCl) and cross-linked hyaluronic acid (xHyA) gels, has been proposed to enhance the efficacy of NSPT. **Objective:** This systematic review aimed to evaluate the clinical effectiveness of the subgingival application of NaOCl and xHyA gels as adjunctive therapies to NSPT in patients with periodontal disease. **Materials and Methods:** A comprehensive literature search was conducted in the MEDLINE (PubMed), Cochrane Library, Web of Science, and Scopus databases following PRISMA guidelines. The review was registered in PROSPERO (CRD420251074045). Randomized clinical trials (RCTs) in human subjects with a follow-up of at least 6 months were included if they assessed outcomes such as PPD, CAL, BOP, or radiographic bone loss (RBL). Studies involving the adjunctive use of NaOCl and xHyA gels were selected according to the PICOS strategy. **Results:** Two RCTs published between 2023 and 2024, with follow-ups ranging from 6 to 9 months and involving 48–50 patients, met the inclusion criteria. Both studies demonstrated significant improvements in clinical outcomes when sodium hypochlorite and hyaluronic acid were used adjunctively with NSPT compared to when NSPT was used alone. Sites treated with adjunctive therapy showed significantly greater reductions in PPD and greater CAL gains over time. Pocket closure rates were also markedly higher in deep sites (>7 mm) in the adjunctive group than in the control group, indicating a substantial regenerative potential and a possible reduction in the need for surgical intervention. Gingival recession exhibited more favorable recovery trends in the adjunctive group, while BOP frequency decreased in both groups without statistically significant differences. **Conclusions:** The adjunctive use of NaOCl and xHyA gels in non-surgical periodontal therapy significantly enhances clinical outcomes compared with the use of mechanical debridement alone.

## 1. Introduction

Periodontal disease is a multifactorial chronic inflammatory disease. It is caused by dysbiosis of the dental biofilm, which leads to the formation of an inflammatory infiltrate that promotes the destruction of the connective tissue attachment supporting the teeth, as well as alveolar bone resorption, potentially culminating in tooth loss [1,2,3,4].

Approximately 90% of the global population may be suffering from extremely common periodontal diseases [5]. At present, one of the most prevalent diseases impacting humankind is periodontitis [5,6], with a concerning 743 million documented cases, ranking sixth in terms of prevalence, according to the Global Burden of Disease Study [7].

According to the National Health and Nutrition Examination Survey (NHANES) conducted between 2009 and 2012, 46% of adults aged 30 or older in the United States suffer from periodontitis, translating to approximately 64.7 million individuals, 8,9% of whom have severe periodontitis. The prevalence tends to increase with age, impacting over 85% of individuals older than 65 [8,9]. Likewise, in Europe, it is reported that roughly 10% of people have suffered from severe periodontal diseases, with the majority of cases occurring in adulthood [10,11,12].

Periodontitis is characterized by the alteration of healthy subgingival plaque with subsequent inflammation and tissue destruction [13,14].

The main contributory factor to periodontitis occurs with the existence of dysbiotic bacterial communities arranged in matrices of extracellular polysaccharides forming subgingival biofilms that adhere to the root surface whilst exposed to the external environment [15,16]. From a contrasting perspective, periodontal health is related to microbial communities in symbiosis with the host [15].

The hundreds of microorganisms that constitute the human oral microbiome colonize a broad variety of surfaces and are mostly organized in complex biofilms [17]. At least 800 bacterial species exist in dental plaque [18,19,20,21,22,23], yet only 54% of these species have been officially identified, 14% have been cultivated, and 32% are uncultivated phylotypes [24].

The main clinical manifestations of periodontitis include the loss of periodontal supporting tissue, translating into clinical attachment loss (CAL) and radiographic bone loss (RBL), with the presence of gingival bleeding and periodontal pockets [4].

Recent guidelines for the treatment of periodontitis stages I-III state that non-surgical periodontal therapy (NSPT) primarily aims to restore periodontal health in the supporting tissues by focusing on reducing and eliminating biofilms and subgingival calculus, decreasing pocket probing depths (PPDs) and bleeding on probing (BOP), and increasing clinical attachment levels through subgingival instrumentation using the mechanical action of curettes combined with ultrasonic devices. The efficacy of subgingival instrumentation can be enhanced by the adjunctive application of chemical agents [25,26,27,28,29,30].

The goal of NSPT is to reduce the pocket-probing depth (PPD) to 4 mm or less and minimize periodontal inflammation with the intention of pocket closure. Thus, for long-term periodontal stability, continuous supportive periodontal therapy (SPT), along with repeated instrumentation as advised by the European Federation of Periodontology (EFP) guidelines, is essential [25,31,32].

### 1.1. Sodium Hypochlorite

Sodium hypochlorite has been widely used as a surface disinfectant. The World Health Organization (WHO) advises hospitals to utilize disinfectants, such as sodium hypochlorite, as part of proper environmental cleaning and disinfection protocols [33].

In endodontic treatment, sodium hypochlorite (NaOCl) is the most used irrigant. Its antimicrobial and tissue-dissolving abilities make it the gold standard of root canal irrigants [34,35].

In vitro and clinical studies have demonstrated that sodium hypochlorite/amino acid gel acts as an antiseptic and is particularly effective against Gram-negative species associated with periodontitis, showing the ability to alter the biofilm matrix [36,37,38].

Laboratory studies have shown that sodium hypochlorite/amino acid gel has a softening effect on the extracellular matrix of biofilms. Subgingival mechanical instrumentation and the chemical reaction of sodium hypochlorite act synergistically in the disruption of the biofilm and the removal of granulation tissue [37,38].

It is important to emphasize that the use of sodium hypochlorite in conjunction with mechanical debridement does not negatively affect the dentin or root cementum. The high pH of sodium hypochlorite interacts with the calculus, promoting its softening, making subgingival mechanical instrumentation easier to perform [38].

### 1.2. Hyaluronic Acid

It is noteworthy that hyaluronic acid is the main component of the extracellular matrix of the joints, skin, and eyes, among other tissues and organs [39]. Numerous in vitro studies have demonstrated that hyaluronic acid accelerates blood clot formation, promotes angiogenesis, and stimulates the osteogenesis process [40,41,42,43,44].

Hyaluronic acid plays a fundamental role in every phase of tissue healing by stimulating the proliferation of cells, as well as their differentiation and migration [39].

The application of natural and cross-linked high molecular weight hyaluronic acid gels during subgingival instrumentation has been considered a favorable option in periodontal therapy [36]. Some authors have reported that they promote better sealing of the supporting tissues surrounding the teeth, exert a bacteriostatic effect on bacterial strains associated with periodontitis, and are beneficial in reducing bacterial contamination [39,45].

To improve the outcomes of NSPT, adjunctive periodontal treatment has recently been proposed, combining the use of sodium hypochlorite/amino acids (Perisolv, Regedent; Zürich, Switzerland) and xHyA (high-molecular) gels (Hyadent BG, Regedent) with subgingival instrumentation [32,46,47,48,49,50].

### 1.3. Objective

The objective of this systematic review was to analyse the scientific literature on the clinical outcomes obtained after the subgingival application of sodium hypochlorite and hyaluronic acid in conjunction with non-surgical periodontal therapy.

## 2. Materials and Methods

### 2.1. Research Conduct

This systematic review was conducted between March 2025 and May 2026 by two independent investigators who selected and analyzed articles in accordance with the Preferred Reporting Items for Systematic Reviews and Meta-Analysis (PRISMA) [51] using the MEDLINE (via PubMed), Cochrane Library, Web of Science, and Scopus databases.

The research search strategy used was as follows: ((((((((periodontal disease[MeSH Terms]) OR (gingivitis[MeSH Terms])) OR (periodontitis[MeSH Terms])) OR (peri-implantitis[MeSH Terms])) AND (periodontal therapy[MeSH Terms])) OR (non-surgical therapy[MeSH Terms])) AND (acid hyaluronic[MeSH Terms])) OR (cross-linked hyaluronic acid[MeSH Terms])) AND (sodium hypochlorite[MeSH Terms]). In Scopus, the search was limited to research articles and studies within the fields of medicine and dentistry. The references of eligible articles were also manually searched. 

The articles were analyzed by title, abstract, and full text. The studies included in this review met all the predefined criteria according to the PICOS strategy (“Population”, “Intervention”, “Comparison”, “Outcomes”, and “Study Design”).

A detailed search flowchart is presented in the Section 3.

The study protocol for this systematic review was registered on the International Prospective of Systematic Reviews (PROSPERO), under number CRD420251074045.

### 2.2. Study Selection

The eligibility criteria were organized using the Population, Intervention, Comparison, Outcome, and Study Design (PICOS) method, as follows in Table 1.

### 2.3. Inclusion Criteria

The inclusion criteria corresponding to the PICO questions were articles written in the English language; articles with no year restriction; human studies; randomized clinical trials (parallel or split-mouth design), with a follow-up period of at least 6 months; studies reporting on the application of sodium hypochlorite/amino acid and xHyA acid gels as adjunctives to non-surgical periodontal therapy; and studies reporting on BOP, PPD, CAL, or RBL as outcomes.

### 2.4. Exclusion Criteria

The exclusion criteria were articles without an available abstract, literature reviews, systematic reviews, meta-analyses, case series, unavailable articles, expert opinions, letters to editors, conference abstracts, animal studies, and studies examining combinations with biomaterials (e.g., bone substitutes or membranes) or growth factors (Appendix A).

### 2.5. Screening Method

Screening was performed by two independent examiners (T.I.d.C./F.A.), in accordance with the inclusion and exclusion criteria defined above, and a third reviewer (H.R.-V.) resolved disagreements to ensure intra- and inter-examiner reliability. The Kappa coefficient test applied in this study resulted in almost perfect agreement (0.81–0.99).

**Figure 1:**
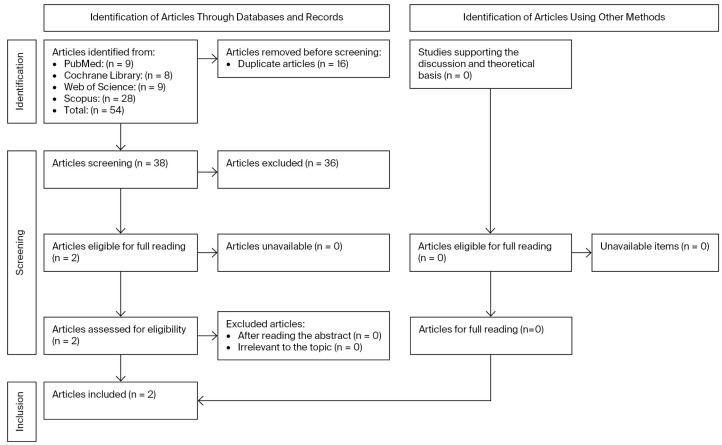
PRISMA 2020 flow diagram.

The process began with the removal of duplicate articles. Titles and abstracts were initially assessed for relevance, followed by an extensive full-text evaluation to ascertain whether the studies sufficiently addressed the research question. The PRISMA 2020 flow diagram is exhibited in Figure 1.

### 2.6. Extraction of Sample Data and Outcomes

The primary outcome was the probing pocket depth (PPD), measured in millimeters from the gingival margin to the bottom of the probed pocket.

The secondary outcomes were the values of variables associated with periodontitis, such as CAL, BOP, GR, PI, FMPI, and FMBOP.

The data were extracted by one reviewer (T.I.C) and summarized in a results table. The information was collected taking into consideration the author, study design and objective, eligibility criteria, study population (sample size and average age group), duration of the study and follow-up period in months, outcome measurement, and results.

### 2.7. Study Quality, Characteristics, and Risk of Bias

To assess the studies’ methodological quality and determine the extent to which they addressed the possibility of bias in their design, conduct, or analysis, two researchers (T.I.C./H.V.) assessed the sample according to the criteria of RoB 2 (randomized clinical trials) tools, which follow the recommendations of the Cochrane Handbook for Systematic Reviews.

## 3. Results

In total, 54 articles were initially identified. After excluding 16 duplicate articles, 38 studies were screened, culminating in 2 articles fulfilling the selection criteria, which were read in full and included in the qualitative synthesis. The Kappa coefficient test applied in this study resulted in almost perfect agreement (0.81–0.99).

### 3.1. Studies Characteristics

The two articles included were randomized clinical trials (RCTs), for which their characteristics are displayed in Table 2. Both studies, published between 2023 [48] and 2024 [50], compared the clinical outcomes of subgingival instrumentation alone and subgingival instrumentation adjunctively used sodium hypochlorite/amino acid and xHyA gels combined together. The follow-up time ranged between 6 [48] and 9 months [50].

The sample sizes were comparable across studies, comprising a total of 98 patients, ranging from 48 [48] to 50 patients [50]; the minimum mean age was 29, and the maximum age was 82.

This systematic review included the assessment of 5344 sites—1448 sites evaluated in the 2024 study by Benyei et al. and 3896 sites evaluated in the 2023 study by Ramanauskaite et al., which required further periodontal treatment [48,50].

### 3.2. Adjuvant Characteristics

This review included two articles [48,50] describing the adjunctive use of a sodium hypochlorite/amino acid gel (Perisolv^®^, Regedent AG, Zürich, Switzerland) administered into the pockets before subgingival instrumentation and repeatedly applied during the procedure.

Subsequently, a mixture of natural and xHyA (high molecular) gel (Hyadent^®^ BG, Regedent AG, Zürich, Switzerland) was delivered inside the pockets up to the gingival margin.

### 3.3. Output Measurement Methods and Results (Table 3)

#### 3.3.1. Pocket Probing Depth (PPD)

Pocket probing depth (PPD) was reported in both studies [48,50]. In the 2024 study by Benyei et al., the PPD means did not significantly differ between the control and test groups at baseline (T1: 4.69 mm, SD = 1.01 mm vs. 4.74 mm, SD = 0.99 mm) [50]. Furthermore, in the 2023 study by Ramanauskaite et al., no significant differences were observed at baseline between the control and test groups in terms of the mean moderate pockets (4–6 mm; T0: 4.8 mm, SD = 0.2 mm and 4.7 mm, SD = 0.2 mm, respectively; *p* = 0.417) and in mean deep pockets (≥7 mm; T0: 8.0 mm, SD = 0.7 mm and 8.2 mm, SD = 0.9 mm, respectively; *p* = 0.443) [48].

As the study progressed, Benyei et al. observed a notable and significant reduction in PPD in both groups (T2: 3.35 mm, SD = 1.08 mm in control group and 3,50 mm, SD = 1.03 mm in test group; T3: 3.14 mm, SD = 1.08 mm and 2.94 mm, SD = 0.82 mm, respectively). Furthermore, the test group showed a considerably greater reduction than the control group at both the 3- and 9-month follow-ups, with that at the latter being statistically significant [50]. Ramanauskaite et al. reported statistically significant improvements in mean moderate pockets and mean deep pockets in both groups at 3 and 6 months compared to baseline (*p* < 0.001); however, statistically significantly higher reductions in PPD were observed at both follow-ups, favoring the test group (*p* < 0.001). The change in PPD between 3 and 6 months significantly differed between the groups, favoring the test group (*p* = 0.002) in mean moderate pockets; however, it did not differ between the groups (*p* = 0.096) in mean deep pockets [48].

#### 3.3.2. Clinical Attachment Level (CAL)

Benyei et al. assessed clinical attachment levels (CALs) and reported no significant differences in the mean values between the groups at baseline (T1: 6.07 mm, SD = 1.59 mm for the control group and 5.85 mm, SD = 1.42 mm for the test group, *p* = 0.105) [50]. However, Ramanauskaite et al. reported that the CAL values at baseline were marginally higher in the control group (4.8 mm, SD = 0.3 mm) than in the test group (4.6 mm, SD = 0.2 mm; *p* = 0.026) in mean moderate pockets but were not statistically significantly different (7.9 mm, SD = 0.6 mm in the control group and 8.1 mm, SD = 0.7 mm in the test group; *p* = 0.412) in mean deep pockets [48].

Nevertheless, as the intervention continued, the study by Benyei et al. found that Group A (the test group) exhibited a superior improvement in CAL compared to Group B (the control group) at both the 3- and 9-month follow-ups (T2: 3.35 mm, SD = 1.08 mm in control group and 3.50 mm, SD = 1.03 mm in the test group; T3: 3.14 mm, SD = 1.01 mm and 2.94 mm, SD = 0.82 mm, respectively), with statistically significant differences (*p* = 0.001) [50]. In addition, Ramanauskaite et al. found statistically significant improvements in both groups at both follow-ups compared to baseline (*p* < 0.001); however, statistically significantly higher values were observed in the test group (*p* < 0.001), even though the mean CAL change between the 3- and 6-month follow-ups did not significantly differ between the groups (*p* = 0.077) [48].

#### 3.3.3. Bleeding on Probing (BOP)

Benyei et al. found that bleeding on probing (BOP) did not significantly differ between the groups at any follow-up (T1—baseline; T2—3 months; T3—9 months), with *p*-values of 0.796, 0.175, and 0.339, respectively [50].

Ramanauskaite et al. evaluated the BOP values of treated sites (PPD ≥ 4 mm), revealing no statistically significant difference in baseline values between the control and test groups (*p* = 0.687). However, both groups demonstrated statistically significant improvements at the 3- and 6-month follow-ups in comparison to baseline (*p* < 0.001). Additionally, the BOP reduction was significantly greater in the test group than in the control group at both the 3- and 6-month follow-ups (*p* = 0.0018 and *p* < 0.001, respectively) [48].

#### 3.3.4. Approximal Plaque Index (API)/Plaque Index (PI)

Benyei et al. presented a graphic illustrating that the development of the approximal plaque index (API) reduced throughout the follow-up periods (T1—baseline; T2—3 months; T3—9 months) [50].

Moreover, Ramanauskaite et al. found that, at baseline, the plaque index (PI) values of treated pockets (PPD > 4 mm) were higher in the test group than in the control group (T0: 60.6%, SD = 10.9% and 38.8%, SD = 26%, respectively, *p* = 0.002). In addition, both study groups showed statistically significant improvements at 3 months (T2: 18.8%, SD = 11.4% in the test group and 20.3%, SD = 16.7% in the control group, *p* = 0.714) and at 6 months (T3: 12.7%, SD = 8.9% in the test group and 26.5%, SD = 20.5% in the control group, *p* = 0.018) compared to baseline (*p* < 0.001). No statistically significant difference was observed between the groups at the 3-month follow-up (*p* = 0.714); however, at the 6-month follow-up, the PI reduction was significantly greater in the test group (*p* = 0.018) [48].

#### 3.3.5. Gingival Recession (GR)/Recession (REC)

Benyei et al. found that the gingival recession (GR) values significantly differed between the groups at baseline (1.38 mm, SD = 1.14 mm in the control group and 1.12 mm, SD = 0.95 mm in the test group, *p* < 0.001). Furthermore, GR showed greater recovery in the test group, which exhibited slight positive changes (T2: 0.95 mm, SD = 0.88 mm; T3: 0.81 mm, SD = 0.82 mm), than in the control group, which showed little progression from baseline to the 3- and 9-month follow-ups (T2: 1.51 mm, SD = 1.15 mm; T3: 1.48 mm, SD = 1.15 mm) [50].

Ramanauskaite et al. took the distance (in millimeters) from the gingival margin to the cemento-enamel junction or to the margin of a cervical restoration as a measurement of recession (REC); however, they only presented clinical attachment level (CAL) values, which were calculated by adding the probing depth (PPD) and recession (REC) at each site [48].

#### 3.3.6. Full Mouth Plaque Index (FMPI)

Ramanauskaite et al. found that the FMPI values were higher in the test group (52.9%, SD = 11.4%) than in the control group (35.7%, SD = 23.7%). Both groups showed significant improvements at the 3- and 6-month follow-ups compared to baseline (*p* < 0.001); however, there was a statistically significant difference between the groups, in favor of the test group at 6 months (*p* = 0.006) [48].

#### 3.3.7. Full Mouth Bleeding on Probing (FMBOP)

Ramanauskaite et al. found that, at baseline, the FMBOP values were similar between the test group (76.5%, SD = 18.2%) and the control group (68.9%, SD = 20.3%): *p* = 0.184. Both study groups exhibited significant improvements at both 3 months (33.3%, SD = 13.7% in the control group and 25.9%, SD = 12.3% in the test group, *p* = 0.06) and 6 months (40.8%, SD = 13.8% in the control group and 15.6%, SD = 9.9% in the test group, *p* < 0.001). The difference between the groups at 3 months was not statistically significant; however, there was a statistically significant difference at the 6-month follow-up, in favor of the test group (*p* < 0.001) [48].

### 3.4. Risk of Bias Assessment

The risk of bias (Table 4) was assessed using the ROB2 tool for two RCTs, which were concealed as “Low risk” [48,50]. After re-evaluating the studies, we found that the results remained clear and did not raise any significant issues. Nonetheless, only one domain (D4) related to outcome measurement was rated as “Some concerns” due to the fact that outcome assessors were likely to be aware of the status of the intervention and were not blinded; however, the outcomes were not influenced by knowledge of the intervention received [50].

Therefore, both studies classified as “Low risk” overall reinforced the investigation value.

## 4. Discussion

The aim of this systematic review was to investigate the scientific literature and the current evidence regarding the clinical outcomes obtained with the subgingival application of sodium hypochlorite and hyaluronic acid in conjunction with non-surgical periodontal therapy. The fact that only two RCTs could be included in our review indicates that that there is scarce clinical evidence available in the scientific literature. Based on our findings, it is determined that adjunctive subgingival application of sodium hypochlorite/amino acids and xHyA can enhance the clinical outcomes of non-surgical periodontal therapy, as demonstrated by improvements in PPD, CAL, and BOP values [48,50].

Periodontitis is a chronic multifactorial inflammatory disease of microbial etiology that results from a dysbiotic shift in the subgingival biofilm and a subsequent destructive host immune–inflammatory response. This interplay between pathogenic microbial communities and the host’s immune mechanisms leads to the progressive loss of the tooth-supporting apparatus, clinically characterized by the formation of periodontal pockets, attachment loss, and radiographic alveolar bone resorption. The disease is modulated by genetic, systemic, and environmental factors—such as smoking, diabetes, and immune dysregulation—that influence both microbial composition and host susceptibility. Several Gram-negative anaerobic bacterial strains have been identified in the biofilms associated with periodontitis. Their roles in the dysbiosis associated with periodontitis have been extensively studied. Some of the most studied bacterial strains are *A. actinomycetemcomitans*, *P. intermedia*, *P. gingivalis*, *T. forsythia*, *T. denticola*, and *F. nucleatum* [52,53,54,55].

For instance, preclinical studies have shown that sodium hypochlorite/amino acid gel effectively disrupts the biofilm matrix and exhibits potent antiseptic activity, particularly against Gram-negative bacterial species associated with periodontitis [36,37,48,50]. Moreover, clinical studies have indicated that the adjunctive use of sodium hypochlorite/amino acid gel provides significant therapeutic benefits in the management of deep periodontal pockets in untreated periodontitis, as well as in residual periodontal pockets, peri-implant mucositis, and peri-implantitis [38,46,47,56,57,58].

Furthermore, hyaluronic acid has been shown to be efficient in reducing the proliferation of bacteria on surgical wounds and to exhibit antibacterial effects on bacterial strains linked to periodontitis [45,49]. In addition to being considered biocompatible with periodontal tissues, preclinical studies on xHyA have indicated that this formulation improves the migration, proliferative, and wound-healing capabilities of the cells essential for soft tissue wound healing [47,59]. Moreover, xHyA can sustain the stemness of osteoprogenitors while strongly promoting their growth, which could potentially stabilize the ratio between differentiation and self-renewal during bone regeneration [47,60].

As demonstrated in the studies by Ramanauskaite et al. and Benyei et al., both mechanical debridement alone and subgingival debridement in combination with xHyA and sodium hypochlorite/amino acids significantly altered microbial activity; however, distinct differences were observed between the test and control groups [47,50]. Both groups displayed statistically significant reductions in *T. forsythia*, *T. denticola*, *P. gingivalis*, and *P. intermedia* at 3 months compared to baseline (*p* < 0.05); nonetheless, the adjunctive group displayed a statistically significant decrease in *P. intermedia* and *P. gingivalis* (*p* < 0.05). However, it is important to emphasize that a statistically significant reduction in A. *actinomycetemcomitans* was only observed in the test group (*p* = 0.001) at 3 months. At 6 months, the detection frequency of *T. forsythia* and *P. gingivalis* statistically significantly decreased in the control group (*p* < 0.01) compared to baseline. However, in the test group, statistically significant reductions were observed for all examined periopathogenic species compared with baseline (*p* < 0.05) [47].

Beyond that, a series of investigations have pointed out that statistically significant decreases in *P. gingivalis*, *T. forsythia*, and *T. denticola* may be a hallmark of effective periodontal therapy [61].

Recent research investigations have demonstrated that sodium hypochlorite/amino acids and xHyA gels can be used adjunctively to treat deep persistent pockets non-surgically while improving clinical parameters efficiently. This allows for the delay or even circumvention of invasive surgical intervention [32,48,49,50,62].

Sodium hypochlorite/amino acid (A2H) gel makes it easier to distinguish between healthy and granulated tissues, decreases the bacterial population by dissolving the biofilm matrix, restricts tissue recession by improving clinical attachment levels (CALs) and decreasing pocket probing depth (PPDs), helps to decontaminate the periodontal sites, and successfully treats the biofilm, as well as being harmless to the tissues and tooth surface when used at a 0.5% concentration [32,47,48,49,50,57,63,64].

Cross-linked hyaluronic acid (high-molecular) gel promotes soft tissue and bone regeneration, accelerates pocket closure by maintaining blood clots, presents bacteriostatic characteristics that promote healing and maintain wound decontamination, and fosters periodontium cell adherence and regeneration, thereby reducing pocket probing depths (PPDs) and improving clinical attachment levels (CALs) [32,46,47,48,49,59,60,61,62,63,64,65,66,67,68,69].

According to the results obtained, adjunctive non-surgical periodontal treatment with sodium hypochlorite/amino acids and xHyA (high molecular) gels has advantages over SRP alone. Ramanauskaite et al. conducted a 6-month study and investigated PPD as the primary outcome. They found that both adjunctive treatment and SRP alone provided statistically significant improvements in every clinical parameter evaluated; however, the combination of sodium hypochlorite/amino acids and xHyA gels with subgingival mechanical debridement yielded statistically significantly better improvements than SRP alone, thereby providing a useful strategy to enhance the clinical outcomes of non-surgical periodontal therapy, such as PPD, CAL, and BOP [48]. Benyei et al. conducted a 9-month study and investigated CAL as the primary outcome. They found that the adjunctive combination of sodium hypochlorite/amino acids and xHyA gels with subgingival mechanical debridement sufficiently improved clinical outcomes, namely, PPD, CAL, and BOP. Furthermore, at the 9-month follow-up, they also found that adjunctive treatment might indicate a propensity for a regenerative response to treatment [50].

Interestingly, both studies reported high efficacy of the adjunctive treatment related to deep pockets. Ramanauskaite et al. found that the number of deep pockets reduced from 277 to 35 in the control group; however, much better results were found in the test group, where the number reduced from 298 to 4 (*p* = 0.003) [48]. These findings are also consistent with those of the study conducted by Benyei et al., who reported a deep pocket closure rate of 94% in the test group, highly contrasting with the rate of 42% in the control group [50].

Another study conducted by Ramanauskaite et al., which included twenty-one systemically healthy patients diagnosed with stage II-III grade A/B periodontitis, also reported that the use of sodium hypochlorite/amino acids and xHyA gels adjunctive to subgingival mechanical debridement may constitute an advantageous method for enhancing the clinical outcomes of NSPT. At 3- and 6-month follow-ups, statistically significant mean reductions in PPD were observed compared to baseline (*p* < 0.001), and the difference between the 3- and 6-month follow-ups was also statistically significant (*p* = 0.004). Furthermore, the mean CAL enhancement was statistically significant at both the 3- and 6-month follow-ups compared to baseline (*p* < 0.001), and a statistically significant difference in CAL gain was also found between the 3- and 6-month follow-ups (*p* = 0.016). A notable and significant difference was found in this study, with a reduction in the number of deep pockets from 319 to 3 [49], which is consistent with the results of Ramanauskaite et al. and Benyei et al. [48,50].

In line with our study, in 2022, Diehl et al. performed a 6-month study in 29 patients with 111 sites receiving treatment. Bleeding on probing decreased by more than 60%, and an overall PPD reduction exceeding 2 mm was achieved, alongside a comparable CAL gain of 2.02 mm. Pocket closure was observed in nearly 25% of sites. Furthermore, they also reported that sodium hypochlorite cleaning–gel application may provide additional benefits to NSPT by enhancing mechanical biofilm removal, thereby amplifying the impact of xHyA. This study concluded that the adjunctive application of sodium hypochlorite and hyaluronic acid gels in NSPT is extremely advantageous [32], a conclusion identical to that of the previously mentioned studies [47,48,49,50].

Another clinical investigation conducted in 2024 by Shirakata et al. [70] compared the histological aspects of NSPT both with and without the adjunctive use of sodium hypochlorite/amino acids and xHyA gels in dogs. The clinical outcomes of this animal study are consistent with those of clinical investigations that concluded that the use of SRP with sodium hypochlorite/amino acids and xHyA gels produced statistically significant enhancements in clinical outcomes, reflected in a decline in BOP values, improvements in CALs, and reductions in PPDs, when compared to baseline or SRP alone [70], which is also in accordance with our study [32,47,48,49,50]. Consistent with the clinical findings, the histological evaluation provided noteworthy evidence supporting these outcomes. The test group showed statistically significantly greater amounts of new connective attachment, new cementum formation, and new bone formation than the control group [70].

To date, only positive results have been obtained regarding the adjunctive use of sodium hypochlorite/amino acids and xHyA (high molecular) gels in non-surgical periodontal therapy (NSPT).

## 5. Conclusions

The adjunctive use of sodium hypochlorite/amino acids and xHyA (high-molecular) gels in non-surgical periodontal therapy (NSPT) significantly enhances clinical outcomes compared to the use of subgingival instrumentation alone. Adjunctive periodontal therapy appears to contribute to a regenerative healing response. Despite these promising results, the limited number of studies highlights the need for further multicenter, long-term randomized controlled trials with longer follow-up periods and larger sample sizes in order to corroborate these findings.

## Figures and Tables

**Table 1:** PICOS strategy.

Question	Is there a relationship between the recovery of periodontal tissues with the application of hyaluronic acid and sodium hypochlorite in the non-surgical approach to periodontal treatment?
P (Population)	Patients with periodontal disease
I (Intervention)	Application of hyaluronic acid and sodium hypochlorite as adjunctives in the non-surgical periodontal therapy
C (Comparison)	The same non-surgical periodontal treatment without application of any adjuvant
O (Outcomes)	Periodontal parameters (BOP, PPD, CAL, RBL)
S (Study Design)	Clinical trials and randomized controlled trials

**Table 2:** Study characteristics.

Author	Study Design	Study Aim	Inclusion Criteria	Exclusion Criteria	Sample Characteristics	Follow-Up	Analyzed Parameters
Benyei L.	RCT	To compare the clinical outcomes obtained in persistent periodontal pockets during a 9-month follow-up of supportive periodontal step 4 treatment performed by either combining subgingival instrumentation with adjunctively used sodium hypochlorite/amino acid gel and cross-linked hyaluronic acid gel (xHyA) or using subgingival instrumentation alone	Systematically, healthy adult individuals who were previously diagnosed with periodontitis according to the clinical practice guideline, who had completed steps 1 and 2 of therapy initially presenting with untreated periodontitis at stage 3 or 4	- Individuals with unregulated T2DM with HbA1c scores > 7.5% - Chronic conditions such as rheumatoid arthritis - Pregnancy/lactating	50 stage 3 and 4 periodontitis patients (20 females and 30 males) Control group (group B): SRP alone (n = 25 [14 females and 11 males]) with mean age of 56.4 [SD = 13.5] Test Group (group A): SRP + adjunctive protocol (n = 25 [7 females and 18 males]) with mean age 60.6 [SD = 11.3]	9 months (T1—baseline; T2—after 3 months; T3—after 9 months)	Primary outcome:CALSecondary outcomes:GRPPDBOP
Ramanauskaite E.	RCT	To compare the clinical outcomes obtained after performing mechanical subgingival debridement in conjunction with sodium hypochlorite and amino acids-containing gel, followed by subsequent application of a cross-linked hyaluronic acid gel (xHyA) with mechanical debridement alone.	- Males and females ≥ 18 years old - Periodontitis stages II-III, grades A/B, generalised - Good general health (i.e., absence of systemic diseases and no intake of medication which might affect periodontal health) - Presence of at least 20 teeth (wisdom teeth excluded) - Absence of removable dentures - Patients willing to provide written informed consent and willing to complete the 6-month follow-up study	- Patients already included in other clinical trials - Smokers - Periodontal treatment during the last 12 months - Antibiotic treatment 3 months prior to the start of the trial - Antibiotic prophylaxis required for dental treatment - Ongoing medication that may affect the clinical features of periodontitis - Pregnant/lactating - Allergies to sodium hypochlorite	48 stage II-III periodontitis patients (13 males and 35 females) Control Group: SRP alone (n = 24 [7 males and 17 females]) with mean age 49.3 [SD = 11.2] Test Group: SRP + NaOCl + HA gels ([6 males and 18 females]) with mean age 47.3 [SD = 10.7]	6 months (T0—baseline; T1—after 3 months; T2—after 6 months)	Primary Outcome: PPD Secondary Outcomes: CAL PI BOP FMPI FMBOP

**Table 3:** Studies outcomes.

Study	Follow-Up	Group	Periodontal Parameters		
			PPD [SD] (mm)	CAL [SD] (mm)	BOP [SD] (%)
Benyei L. 2024	Baseline (T1)	Control	4.69 [1.01]	6.07 [1.59]	0.78 [0.42]
		Test	4.74 [0.99]	5.85 [1.42]	0.78 [0.42]
	3 Months (T2)	Control	3.35 [1.08]	4.85 [1.66]	0.21 [0.41]
		Test	3.50 [1.03]	4.44 [1.37]	0.17 [0.48]
	9 Months (T3)	Control	3.14 [1.01]	4.59 [1.70]	0.13 [0.34]
		Test	2.94 [0.82]	3.76 [1.18]	0.11 [0.32]
Ramanauskaite E. 2023	Baseline (T0)	Control	MP:4.8 [0.2]	MP:4.8 [0.3]	81.8 [16.2]
			DP:8.0 [0.7]	DP:7.9 [0.6]	
		Test	MP:4.7 [0.2]	MP:4.6 [0.2]	83.2 [15.5]
			DP:8.2 [0.9]	DP:8.1 [0.7]	
	3 Months (T1)	Control	MP:2.9 [0.7]	MP:3.1 [0.8]	39.1 [15.9]
			DP:4.4 [1.4]	DP:4.5 [1.2]	
		Test	MP:2.2 [0.4]	MP:2.4 [0.6]	28.3 [14.6]
			DP:2.9 [0.1]	DP:3.2 [1.4]	
	6 Months (T2)	Control	MP:3.0 [0.6]	MP:3.1 [0.7]	48.9 [14.5]
			DP:4.3 [1.0]	DP:4.6 [1.0]	
		Test	MP:1.8 [0.4]	MP:2.0 [0.5]	17.6 [11.5]
			DP:2.4 [1.0]	DP:2.8 [1.3]	

Legend: MP—moderate pockets (4–6 mm); DP—deep pockets (≥7 mm).

**Table 4:** The risk of bias assessment.

Study	Design	ROB Tool	D1	D2	D3	D4	D5	Overall
Benyei L. 2024	RCT	ROB 2	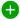	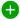	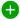	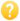	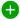	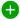
Ramanauskaite E. 2023	RCT	ROB 2	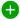	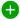	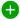	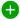	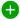	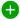

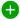
 Low risk; 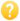 some concerns. ROB2 domains—D1, randomization process; D2, deviations from the intended interventions; D3, certain outcome data; D4, measurement of the outcome; D5, selection of the reported result.

## Data Availability

The original contributions presented in this study are included in the article. Further inquiries can be directed to the corresponding author.

## Abbreviations

The following abbreviations are used in this manuscript:

GR	Gingival recession;
PPD	Pocket probing depth;
CAL	Clinical attachment level;
BOP	Bleeding on probing;
PI	Plaque index;
FMPI	Full mouth plaque index;
FMBOP	Full mouth bleeding on probing;
REC	Recession;
xHyA	Cross-linked hyaluronic acid gel;
NaOCl	Sodium hypochlorite;
NSPT	Non-surgical periodontal therapy;
SRP	Scaling and root planning.

## Appendix A

**Table A1:** Exclusion criteria per article—(1) reviews and clinical cases; (2) in vitro and animals; (3) combinations with biomaterials or growth factors; (4) non-published studies; (5) follow-up < 3 months; (6) other science or thematic; (7) sodium hypochlorite or hyaluronic acid used separately.

Author	Year	Title	Criteria
P. J. Palma; J. C. Ramos; J. B. Martins; A. Diogenes; M. H. Figueiredo; P. Ferreira; C. Viegas; J. M. Santos	2017	Histologic Evaluation of Regenerative Endodontic Procedures with the Use of Chitosan Scaffolds in Immature Dog Teeth with Apical Periodontitis	2 + 6
D. Diehl; A. Friedmann; P. Liedloff; R. M. Jung; A. Sculean; H. Bilhan	2022	Adjunctive Application of Hyaluronic Acid in Combination with a Sodium Hypochlorite Gel for Non-Surgical Treatment of Residual Pocket Reduces the Need for Periodontal Surgery-Retrospective Analysis of a Clinical Case Series	1
E. Ramanauskaite; V. Machiulskiene; U. M. Dvyliene; M. Eliezer; A. Sculean	2023	Clinical Evaluation of a Novel Combination of Sodium Hypochlorite/Amino Acid and Cross-linked Hyaluronic Acid Adjunctive to Non-surgical Periodontal Treatment: A Case Series	1
D. Irani; G. Jungbauer; A. Sculean; S. Eick	2024	Effect of sodium hypochlorite gel on bacteria associated with periodontal disease	2 + 7
Y. Shirakata; T. Nakamura; F. Setoguchi; T. Imafuji; Y. Shinohara; S. Matsumura; M. Iwata; K. Noguchi; E. Ramanauskaite; A. Sculean	2024	Histological evaluation of nonsurgical periodontal treatment with and without the use of sodium hypochlorit/amino acids and cross-linked hyaluronic acid gels in dogs	2
E. Ramanauskaite; V. M. Visockiene; Y. Shirakata; A. Friedmann; L. Pereckaite; A. Balciunaite; U. M. Dvyliene; A. Vitkauskiene; N. Baseviciene; A. Sculean	2024	Microbiological Effects of Sodium Hypochlorite/-Amino Acids and Cross-linked Hyaluronic Acid Adjunctive to Non-surgical Periodontal Treatment	6
A. Friedmann; R. Jung; H. Bilhan; H. A. Ghawi-Begovic; F. Kauffmann; D. Diehl	2024	Reconstructive surgical therapy of peri-implant defects with ribose cross-linked collagen matrix and crosslinked hyaluronic acid—a prospective case series	1 + 3
L. Ramaglia	2023	Treatment of Peri-implant Mucositis by Sodium Hypochlorite Gel and Cross-linked Hyaluronic Acid Gel	4
D. Diehl	2024	Regenerative potential of the combination of a sodium hypochlorite gel and high molecular weight hyaluronic acid as adjuvants in non-surgical periodontitis therapy—a randomized clinical trial	4
University of Witten/Herdecke	2024	Non-surgical Step 3 Periodontal Treatment With/Without Adjunctive Protocol—Pilot RCT	4
E. Catana	2024	Evaluation of the response to a protocol of treatment for severe periodontitis using subgingival instrumentation plus a combination of sodium hypochlorite and hyaluronic acid	4
K. Ismail	2025	Testing Sodium Hypochlorite and Hyaluronic Acid as na Adjunct to Non-Invasive Therapy for Stage III Periodontitis Management	4
H.A Gloria; M. Nemcová; E. Prikopová; D. Smejkalová; M. Pravda; L. Kucera; V. Velebny	2012	Reductive alkylation of hyaluronic acid for the synthesis of biocompatible hydrogels by click chemistry	6
Fujioka-Kobayashi, M.; Mueller, H.; Mueller, A.; Lussi, A.; Sculean, A.; Schmidlin, P. R.; Miron, R. J.	2017	In vitro effects of hyaluronic acid on human periodontal ligament cells	2 + 7
Mueller, A.; Fujioka-Kobayashi, M.; Mueller, H.; Lussi, A; Sculean, A.; Schmidlin, P. R.; Miron, R. J.	2017	Effect of hyaluronic acid on morphological changes to dentin surfaces and subsequent effect on periodontal ligament cell survival, attachment, and spreading	7
Varghese, J.; Ballal, V.	2018	Antimicrobial efficacy of a denovo anti-plaque agent against periodontal pathogens	6
Madla-Cruz, E.; De la Garza-Ramos, M.; Romo-Sáenz, C. I.; Tamez-Guerra, P.; Garza-Navarro, M. A.; Urrutia-Baca, V.; Martínex-Rodríguez, M. A.; Gomez-Flores, R.	2020	Antimicrobial activity and inhibition of biofilm formation in vitro and on human dentine by silver nanoparticles/carboxymethyl-cellulose composites	6
Mehrabkhani, M.; Parisay, I.; Mastoory, N.; Doghai, V. B.	2021	Effect of casein phosphopeptide amorphous calcium phosphate and xylitol chewing gums, and probiotic yogurt on periodontal parameters: A randomized clinical trial	6
Iorio-Siciliano, V.; Ramaglia, L.; Isola, G.; Blasi, A.; Salvi, G. E.; Sculean, A.	2021	Changes in clinical parameters following adjunctive local sodium hypochlorite gel in minimally invasive nonsurgical therapy (MINST) of periodontal pockets: a 6-month randomized controlled clinical trial	7
Shehatta, O.M.; Osman, A. L.; Elsayed, W. S.; Reddy, S.; Dsouza, J.; Abdelmagyd, H.; Shadroch, D. F.	2022	Antimicrobial Efficacy of Chlorhexidine and Hyaluronic Acid Mouthwashes on Streptococcus Viridans: An In-Vitro Study	2 + 7
Ariel, H.; Kahn, A.; Hila, Z.; Anton, S.; Natan, G.; Kolerman, R.	2022	A thermosensitive gel with an active hyaluronic acid ingredient that contains an octenidine preservation system as na adjunct to scaling and root planning: a randomized prospective clinical study	6
Perelli, M.; Abundo, R.; Semenza, M.; Centracchio, M.; Chiara, S. D.; Monaco, A.; Arduino, P. G.	2022	Preliminary Evaluation of a NitrAdine-Based Brushing Solution for Patients Suffering from Gingivitis: A Prospective Clinical Case-Control Study	1
Ribeiro, J. S.; Sanz, C. K.; Münchow, E. A.; Kalra, N.; Dubey, N.; Suárez, C. E. C.; Fenno, J. C.; Lund, R. G.; Bottino M. C.	2022	Photocrosslinkable methacrylated gelatin hydrogel as a cell-friendly injectable delivery system for chlorhexidine in regenerative endodontics	6
Bertl, K.; Vlachou, S.; Pandis, N.; Zampelis, a.; Stavropoulos, A.	2024	Repeated local delivery of hyaluronic acid gel as adjunctive treatment of residual pockets in periodontitis patients undergoing supportive periodontal care. A randomized controlled clinical trial	7
Portocarrero-Reyes, A.; Asmat-Abanto, A. S.; Ulloa-Cueva, T. V.	2024	Conventional and Herbal Mouthwashes in the Treatment of Periodontal Disease	6
Mišković, I.; Kuiš D.; Špalj, S.; Pupovac, A.; Prpić, J.	2024	Periodontal Health Status in Adults Exposed to Tobacco Heating System Aerosol and Cigarette Smoke vs. Non-Smokers: A Cross-Sectional Study	1
Babina, K.; Salikhova, D.; Makeeva, I.; Zaytsev, A.; Sokhova, I. Musaeva, S.; Polyakova, M.; Novozhilova, N.	2024	A Three-Month Probiotic (the Streptococcus salivarius M18 Strain) Supplementation Decreases Gingival Bleeding and Plaque Accumulation: A Randomized Clinical Trial	6
Ioro-Siciliano, V.; Marasca, D.; Mauriello, L.; Vaia, E.; Stratul, S.; Ramaglia, L.	2024	Treatment of peri-implant mucositis using spermidine and calcium chloride as local adjunctive delivery to non-surgical mechanical debridement: a double-blind randomized controlled clinical trial	6
Meyle, J.; Fischer-Wasels, L.	2024	Non-surgical treatment of peri-implantitis	7
Ioro-Siciliano, V.; Blasi, A.; Mauriello, L.; Salvi, G. E.; Ramaglia, L.; Sculean, A.	2025	Non-Surgical Treatment of Moderate Periodontal Intrabony Deffects with Adjunctive Cross-Linked Hyaluronic Acid: A Single-Blinded Randomized Controlled Clinical Trial	7
Gundogdu Ezer, U.; Gunpinar, S.	2025	Local application of 0.8% hyaluronic acid gel as adjunct to minimally invasive nonsurgical treatment of periodontal intrabony deffects—A randomized clinical trial	7
El Shakhs, R. A. E. A. E. S.; Badran, A. S.; Khattab, N. M. A.	2025	Antibacterial Effect of Hyaluronic Acid Compared to Propylene Glycol as Vehicle for Double Antibiotic paste Against Bacterial Strains in Non-Vital Primary Molar (In Vitro Study)	2
Sales, L.S.; Hewitt, B.; Muchova, M.; Brighenti, F. L.; Kuehne, S. A.; Grant, M. M.; Milward, M. R.	2025	Anti-inflammatory, antioxidant and antimicrobial evolution of morin	6
Al-Abbadi, R.; Shemais, N.; Nawwar, A.; Fawzy El-Sayed, K. M.	2025	Non-surgical periodontal therapy with and without hyaluronic acid gel in type 2 diabetic stage-II periodontitis patients: a randomized clinical trial	7
Saraç Atagün, Ö.; Ceylan-Şen, S.; Ustaoğlu, G.; Özcan, E.	2025	Evaluation of the effects of using an interdental brush dipped in 0.2% hyaluronic acid gel on clinical periodontal parameters among patients with periodontitis: a randomized controlled trial	7
Karkoutly, M.; Abu Hasna, A.; Nam, O. H.; Machado, R.; Al Kurdi, S.; Bshara, N.	2025	Clinical and radiographic outcomes after pulpotomies using mineral trioxide aggregate mixed with distilled water or 2.25% sodium hypochlorite gel: a randomized controlled clinical trial	6

## References

[1] Belibasakis G.N., Belstrøm D., Eick S., Gursoy U.K., Johansson A., Könönen E. (2023). Periodontal microbiology and microbial etiology of periodontal diseases: Historical concepts and contemporary perspectives. Periodontol. 2000.

[2] Borisy G.G., Valm A.M. (2021). Spatial scale in analysis of the dental plaque microbiome. Periodontol. 2000.

[3] Sedghi L., DiMassa V., Harrington A., Lynch S.V., Kapila Y.L. (2021). The oral microbiome: Role of key organisms and complex networks in oral health and disease. Periodontol. 2000.

[4] Papapanou P.N., Sanz M., Buduneli N., Dietrich T., Feres M., Fine D.H., Flemmig T.F., Garcia R., Giannobile W.V., Graziani F. (2018). Periodontitis: Consensus report of workgroup 2 of the 2017 World Workshop on the Classification of Periodontal and Peri-Implant Diseases and Conditions. J. Periodontol..

[5] Pihlstrom B.L., Michalowicz B.S., Johnson N.W. (2005). Periodontal diseases. Lancet.

[6] Papapanou P.N. (2012). The Prevalence of Periodontitis in the US. J. Dent. Res..

[7] Kassebaum N.J., Smith A.G.C., Bernabé E., Fleming T.D., Reynolds A.E., Vos T., Murray C.J.L., Marcenes W., GBD 2015 Oral Health Collaborators (2017). Global, Regional, and National Prevalence, Incidence, and Disability-Adjusted Life Years for Oral Conditions for 195 Countries, 1990–2015: A Systematic Analysis for the Global Burden of Diseases, Injuries, and Risk Factors. J Dent. Res..

[8] Chung S.Y., Song K.B., Lee S.G., Choi Y.H. (2011). The strength of age effect on tooth loss and periodontal condition in Korean elderly. Arch. Gerontol. Geriatr..

[9] Eke P.I., Dye B.A., Wei L., Slade G.D., Thornton-Evans G.O., Borgnakke W.S., Taylor G.W., Page R.C., Beck J.D., Genco R.J. (2015). Update on Prevalence of Periodontitis in Adults in the United States: NHANES 2009 to 2012. J. Periodontol..

[10] Carasol M., Llodra J.C., Fernández-Meseguer A., Bravo M., García-Margallo M.T., Calvo-Bonacho E., Sanz M., Herrera D. (2016). Periodontal conditions among employed adults in Spain. J. Clin. Periodontol..

[11] König J., Holtfreter B., Kocher T. (2010). Periodontal health in Europe: Future trends based on treatment needs and the provision of periodontal services—Position paper 1. Eur. J. Dent. Educ..

[12] Schützhold S., Kocher T., Biffar R., Hoffmann T., Schmidt C.O., Micheelis W., Jordan R., Holtfreter B. (2015). Changes in prevalence of periodontitis in two German population-based studies. J. Clin. Periodontol..

[13] Darveau R.P., Curtis M.A. (2021). Oral biofilms revisited: A novel host tissue of bacteriological origin. Periodontol. 2000.

[14] Jakubovics N.S., Goodman S.D., Mashburn-Warren L., Stafford G.P., Cieplik F. (2021). The dental plaque biofilm matrix. Periodontol. 2000.

[15] Sanz M., Beighton D., Curtis M.A., Cury J.A., Dige I., Dommisch H., Ellwood R., Giacaman R.A., Herrera D., Herzberg M.C. (2017). Role of microbial biofilms in the maintenance of oral health and in the development of dental caries and periodontal diseases. Consensus report of group 1 of the Joint EFP/ORCA workshop on the boundaries between caries and periodontal disease. J. Clin. Periodontol..

[16] Whittaker C.J., Klier C.M., Kolenbrander P.E. (1996). Mechanisms of Adhesion by Oral Bacteria. Annu. Rev. Microbiol..

[17] Filoche S., Wong L., Sissons C.H. (2010). Oral Biofilms: Emerging Concepts in Microbial Ecology. J. Dent. Res..

[18] Paster B.J., Boches S.K., Galvin J.L., Ericson R.E., Lau C.N., Levanos V.A., Sahasrabudhe A., Dewhirst F.E. (2001). Bacterial Diversity in Human Subgingival Plaque. J. Bacteriol..

[19] Paster B.J., Olsen I., Aas J.A., Dewhirst F.E. (2006). The breadth of bacterial diversity in the human periodontal pocket and other oral sites. Periodontol. 2000.

[20] Becker M.R., Paster B.J., Leys E.J., Moeschberger M.L., Kenyon S.G., Galvin J.L., Boches S.K., Dewhirst F.E., Griffen A.L. (2002). Molecular Analysis of Bacterial Species Associated with Childhood Caries. J. Clin. Microbiol..

[21] Aas J.A., Paster B.J., Stokes L.N., Olsen I., Dewhirst F.E. (2005). Defining the Normal Bacterial Flora of the Oral Cavity. J. Clin. Microbiol..

[22] Aas J.A., Griffen A.L., Dardis S.R., Lee A.M., Olsen I., Dewhirst F.E., Leys E.J., Paster B.J. (2008). Bacteria of Dental Caries in Primary and Permanent Teeth in Children and Young Adults. J. Clin. Microbiol..

[23] Preza D., Olsen I., Aas J.A., Willumsen T., Grinde B., Paster B.J. (2008). Bacterial Profiles of Root Caries in Elderly Patients. J. Clin. Microbiol..

[24] Siddiqui R., Badran Z., Boghossian A., Alharbi A.M., Alfahemi H., Khan N.A. (2023). The increasing importance of the oral microbiome in periodontal health and disease. Future Sci. OA.

[25] Sanz M., Herrera D., Kebschull M., Chapple I., Jepsen S., Beglundh T., Tonetti M.S., EFP Workshop Participants and Methodological Consultants (2020). Treatment of stage I-III periodontitis-The EFP S3 level clinical practice guideline. J. Clin. Periodontol..

[26] Ramanauskaite E., Machiulskiene V. (2020). Antiseptics as adjuncts to scaling and root planing in the treatment of periodontitis: A systematic literature review. BMC Oral Health.

[27] Salvi G.E., Mombelli A., Mayfield L., Rutar A., Suvan J., Garrett S., Lang N.P. (2002). Local antimicrobial therapy after initial periodontal treatment. J. Clin. Periodontol..

[28] Smiley C.J., Tracy S.L., Abt E., Michalowicz B.S., John M.T., Gunsolley J., Cobb C.M., Rossmann J., Harrel S.K., Forrest J.L. (2015). Systematic review and meta-analysis on the nonsurgical treatment of chronic periodontitis by means of scaling and root planing with or without adjuncts. J. Am. Dent. Assoc..

[29] Heitz-Mayfield L.J.A., Trombelli L., Heitz F., Needleman I., Moles D. (2002). A systematic review of the effect of surgical debridement vs non-surgical debridement for the treatment of chronic periodontitis. J. Clin. Periodontol..

[30] Heitz-Mayfield L.J.A. (2005). How effective is surgical therapy compared with nonsurgical debridement?. Periodontol. 2000.

[31] Suvan J., Leira Y., Moreno Sancho F.M., Graziani F., Derks J., Tomasi C. (2020). Subgingival instrumentation for treatment of periodontitis. A systematic review. J. Clin. Periodontol..

[32] Diehl D., Friedmann A., Liedloff P., Jung R.M., Sculean A., Bilhan H. (2022). Adjunctive Application of Hyaluronic Acid in Combination with a Sodium Hypochlorite Gel for Non-Surgical Treatment of Residual Pockets Reduces the Need for Periodontal Surgery—Retrospective Analysis of a Clinical Case Series. Materials.

[33] Selam M.N., Tegegne A.M., Ababu A., Matsabisa M., Birhanu G. (2023). Surface Disinfection Practice in Public Hospitals in the Era of COVID-19: Assessment of Disinfectant Solution Preparation and Use in Addis Ababa, Ethiopia. Infect. Drug Resist..

[34] Cai C., Chen X., Li Y., Jiang Q. (2023). Advances in the Role of Sodium Hypochlorite Irrigant in Chemical Preparation of Root Canal Treatment. Biomed. Res. Int..

[35] Haapasalo M., Shen Y., Wang Z., Gao Y. (2014). Irrigation in endodontics. Br. Dent. J..

[36] Costa F.O., Takenaka-Martinez S., Cota L.O.M., Ferreira S.D., Silva G.L.M., Costa J.E. (2012). Peri-implant disease in subjects with and without preventive maintenance: A 5-year follow-up. J. Clin. Periodontol..

[37] Jurczyk K., Nietzsche S., Ender C., Sculean A., Eick S. (2016). In-vitro activity of sodium-hypochlorite gel on bacteria associated with periodontitis. Clin. Oral Investig..

[38] Roos-Jansåker A.M., Almhöjd U.S., Jansson H. (2017). Treatment of peri-implantitis: Clinical outcome of chloramine as an adjunctive to non-surgical therapy, a randomized clinical trial. Clin. Oral Implant. Res..

[39] Olczyk P., Komosińska-Vassev K., Winsz-Szczotka K., Kuźnik-Trocha K., Olczyk K. (2008). Hyaluronan: Structure, metabolism, functions, and role in wound healing. Postepy Hig. Med. Dosw..

[40] Scully M.F., Kakkar V.V., Goodwin C.A., O’Regan M. (1995). Inhibition of fibrinolytic activity by hyaluronan and its alcohol ester derivatives. Thromb. Res..

[41] Deed R., Rooney P., Kumar P., Norton J.D., Smith J., Freemont A.J., Kumar S. (1997). Early-response gene signalling is induced by angiogenic oligosaccharides of hyaluronan in endothelial cells. Inhibition by non-angiogenic, high-molecular-weight hyaluronan. Int. J. Cancer.

[42] de Brito Bezerra B., Mendes Brazão M.A., de Campos M.L.G., Casati M.Z., Sallum E.A., Sallum A.W. (2012). Association of hyaluronic acid with a collagen scaffold may improve bone healing in critical-size bone defects. Clin. Oral Implant. Res..

[43] Kawano M., Ariyoshi W., Iwanaga K., Okinaga T., Habu M., Yoshioka I., Tominaga K., Nishihara T. (2011). Mechanism involved in enhancement of osteoblast differentiation by hyaluronic acid. Biochem. Biophys. Res. Commun..

[44] Sasaki T., Watanabe C. (1995). Stimulation of osteoinduction in bone wound healing by high-molecular hyaluronic acid. Bone.

[45] Jepsen S., Berglundh T., Genco R., Aass A.M., Demirel K., Derks J., Figuero E., Giovannoli J.L., Goldstein M., Lambert F. (2015). Primary prevention of peri-implantitis: Managing peri-implant mucositis. J. Clin. Periodontol..

[46] Iorio-Siciliano V., Ramaglia L., Isola G., Blasi A., Salvi G.E., Sculean A. (2021). Changes in clinical parameters following adjunctive local sodium hypochlorite gel in minimally invasive nonsurgical therapy (MINST) of periodontal pockets: A 6-month randomized controlled clinical trial. Clin. Oral Investig..

[47] Ramanauskaite E., Machiulskiene Visockiene V., Shirakata Y., Friedmann A., Pereckaite L., Balciunaite A., Dvyliene U.M., Vitkauskiene A., Baseviciene N., Sculean A. (2024). Microbiological Effects of Sodium Hypochlorite/-Amino Acids and Cross-linked Hyaluronic Acid Adjunctive to Non-surgical Periodontal Treatment. Oral Health Prev. Dent..

[48] Ramanauskaite E., Machiulskiene V., Shirakata Y., Dvyliene U.M., Nedzelskiene I., Sculean A. (2023). Clinical evaluation of sodium hypochlorite/amino acids and cross-linked hyaluronic acid adjunctive to non-surgical periodontal treatment: A randomized controlled clinical trial. Clin. Oral Investig..

[49] Ramanauskaite E., Machiulskiene V., Dvyliene U.M., Eliezer M., Sculean A. (2023). Clinical Evaluation of a Novel Combination of Sodium Hypochlorite/Amino Acid and Cross-linked Hyaluronic Acid Adjunctive to Non-surgical Periodontal Treatment: A Case Series. Oral Health Prev. Dent..

[50] Benyei L., Friedmann A., Ostermann T., Diehl D. (2024). Non-surgical treatment of residual periodontal pockets using sodium hypochlorite/amino acid gel and cross-linked hyaluronic acid-a 9-month pilot randomized controlled clinical trial. Clin. Oral Investig..

[51] Page M.J., McKenzie J.E., Bossuyt P.M., Boutron I., Hoffmann T.C., Mulrow C.D., Shamseer L., Tetzlaff J.M., Akl E.A., Brennan S.E. (2021). The PRISMA 2020 statement: An updated guideline for reporting systematic reviews. BMJ.

[52] Alghamdi A.S., Almarghlani A.A. (2019). Periodontal pathogenic bacteria among high school children in Saudi Arabia. Ann. Saudi Med..

[53] Costalonga M., Herzberg M.C. (2014). The oral microbiome and the immunobiology of periodontal disease and caries. Immunol. Lett..

[54] Nędzi-Góra M., Kowalski J., Górska R. (2017). The Immune Response in Periodontal Tissues. Arch. Immunol. Ther. Exp..

[55] Stokowa-Sołtys K., Wojtkowiak K., Jagiełło K. (2021). Fusobacterium nucleatum—Friend or foe?. J. Inorg. Biochem..

[56] Megally A., Zekeridou A., Cancela J., Giannopoulou C., Mombelli A. (2020). Short ultrasonic debridement with adjunctive low-concentrated hypochlorite/amino acid gel during periodontal maintenance: Randomized clinical trial of 12 months. Clin. Oral Investig..

[57] Radulescu V., Boariu M.I., Rusu D., Roman A., Surlin P., Voicu A., Didilescu A.C., Jentsch H., Siciliano V.I., Ramaglia L. (2022). Clinical and microbiological effects of a single application of sodium hypochlorite gel during subgingival re-instrumentation: A triple-blind randomized placebo-controlled clinical trial. Clin. Oral Investig..

[58] Iorio-Siciliano V., Blasi A., Stratul S.I., Ramaglia L., Sculean A., Salvi G.E., Rusu D. (2020). Anti-infective therapy of peri-implant mucositis with adjunctive delivery of a sodium hypochlorite gel: A 6-month randomized triple-blind controlled clinical trial. Clin. Oral Investig..

[59] Asparuhova M.B., Kiryak D., Eliezer M., Mihov D., Sculean A. (2019). Activity of two hyaluronan preparations on primary human oral fibroblasts. J. Periodontal Res..

[60] Asparuhova M.B., Chappuis V., Stähli A., Buser D., Sculean A. (2020). Role of hyaluronan in regulating self-renewal and osteogenic differentiation of mesenchymal stromal cells and pre-osteoblasts. Clin. Oral Investig..

[61] Cosyn J., Wyn I., De Rouck T., Sabzevar M.M. (2007). Subgingival Chlorhexidine Varnish Administration as an Adjunct to Same-Day Full-Mouth Root Planing. I. Clinical Observations. J. Periodontol..

[62] Eliezer M., Imber J.C., Sculean A., Pandis N., Teich S. (2019). Hyaluronic acid as adjunctive to non-surgical and surgical periodontal therapy: A systematic review and meta-analysis. Clin. Oral Investig..

[63] Yıldırım S., Özener H.Ö., Doğan B., Kuru B. (2018). Effect of topically applied hyaluronic acid on pain and palatal epithelial wound healing: An examiner-masked, randomized, controlled clinical trial. J. Periodontol..

[64] Schmidlin P.R., Fujioka-Kobayashi M., Mueller H.D., Sculean A., Lussi A., Miron R.J. (2017). Effects of air polishing and an amino acid buffered hypochlorite solution to dentin surfaces and periodontal ligament cell survival, attachment, and spreading. Clin. Oral Investig..

[65] Rajan P., Dusanapudi L., Kumar C., Nair D. (2013). Hyaluronic acid—A simple, unusual polysaccharide: A potential mediator for periodontal regeneration. Univers. Res. J. Dent..

[66] King S.R., Hickerson W.L., Proctor K.G. (1991). Beneficial actions of exogenous hyaluronic acid on wound healing. Surgery.

[67] Pilloni A., Schmidlin P.R., Sahrmann P., Sculean A., Rojas M.A. (2019). Effectiveness of adjunctive hyaluronic acid application in coronally advanced flap in Miller class I single gingival recession sites: A randomized controlled clinical trial. Clin. Oral Investig..

[68] Mueller A., Fujioka-Kobayashi M., Mueller H.D., Lussi A., Sculean A., Schmidlin P.R., Miron R.J. (2017). Effect of hyaluronic acid on morphological changes to dentin surfaces and subsequent effect on periodontal ligament cell survival, attachment, and spreading. Clin. Oral Investig..

[69] Graves D.T., Oates T., Garlet G.P. (2011). Review of osteoimmunology and the host response in endodontic and periodontal lesions. J. Oral Microbiol..

[70] Shirakata Y., Nakamura T., Setoguchi F., Imafuji T., Shinohara Y., Matsumura S., Iwata M., Noguchi K., Ramanauskaite E., Sculean A. (2024). Histological evaluation of nonsurgical periodontal treatment with and without the use of sodium hypochlorite / amino acids and cross-linked hyaluronic acid gels in dogs. Clin. Oral Investig..

